# Potential and Limitations of Atrial Natriuretic Peptide as Biomarker in Pediatric Heart Failure—A Comparative Review

**DOI:** 10.3389/fped.2018.00420

**Published:** 2019-01-29

**Authors:** Tanja Gangnus, Bjoern B. Burckhardt

**Affiliations:** Institute of Clinical Pharmacy and Pharmacotherapy, Heinrich-Heine-University, Düsseldorf, Germany

**Keywords:** pediatric, natriuretic peptide, heart failure, ANP, BNP, sacubitril, preanalytical, saliva

## Abstract

Although B-type Natriuretic Peptide (BNP), N-terminal-proBNP (NT-proBNP), and mid-regional-proANP (MR-proANP) are included in current guidelines on heart failure in adults, no guideline considering these biomarkers in pediatric heart failure is available. A new drug class of neprilysin inhibitors as fixed-dose combination (Sacubitril/valsartan) has been introduced and is currently being investigated in children suffering from heart failure. Atrial Natriuretic Peptide (ANP) is discussed as a more useful alternative to BNP because it may grants better insights into the effects of this treatment. Thus, this review aimed to provide an overview of the current knowledge concerning ANP in pediatric heart failure and compares its suitability regarding diagnosis and prognosis of heart failure. A literature search using PubMed resulted in 147 publications of which 22 studies were classified as relevant. The review presents available ANP, NT-proANP, and MR-proANP level data in children (0–18 years). Summarizing, ANP shows only minor differences as marker for diagnosing and monitoring pediatric heart failure if compared to BNP. Due to its fast release, ANP offers the advantage of displaying rapid changes during therapy or operation. ANP is -like the other natriuretic peptides- influenced by age, presenting with the highest levels in very young infants. ANP also correlates with atrial pressure and volume overload in children. In addition, ANP determination in saliva appears to be a promising alternative to blood sampling. Similarly to NT-proBNP, NT-proANP, and MR-proANP offer better stability but only few data has been published in children and thus their potential is only presumable so far.

## Introduction

Heart failure has been defined as a clinical and pathophysiologic syndrome that results from ventricular dysfunction, volume or pressure overload or a combination of these causes. The inability of the heart to supply the body sufficiently with oxygen results in an activation of the Renin-Angiotensin-Aldosterone-System and the sympathetic nervous system, leading to an upregulation of blood flow. Important counterregulatory hormones are the natriuretic peptides, among which Atrial Natriuretic Peptide (ANP) and B-type Natriuretic Peptide (BNP) play a major role. Both peptides mediate vasodilation, natriuresis, diuresis, and block renin, resulting in a down-regulation of the Renin-Angiotensin-Aldosterone-System (1, 2).

The guidelines published by the European Society of Cardiology (2016) recommend the clinical biomarkers BNP, N-terminal-proBNP (NT-proBNP), and mid-regional-proANP (MR-proANP) for diagnosis and prognosis of heart failure (3). Whereas, these natriuretic peptides are included in guidelines for adults, there is no guide considering BNP, NT-proBNP, and MR-proANP in pediatric heart failure. Different etiology in adults and children, the effect of ontogeny on clinical course and outcome are some of the reasons why recommendations given in the adult guidelines cannot be simply transferred to pediatrics (4).

The recently introduced drug class of neprilysin inhibitors to patients suffering from heart failure raised the question whether ANP is the better alternative compared to BNP to adequately reflect effects during therapy with sacubitril/valsartan (Entresto®) (5). ANP is more susceptible to degradation by neprilysin than BNP. Changes effected by this treatment may be more obvious than the observed variation of BNP and NT-proBNP, which was within the weekly fluctuation (6). Currently sacubitril/valsartan is being investigated in children suffering from heart failure within the PANORAMA-HF study (7).

This review provides an overview outlining the current knowledge on pediatric ANP levels focusing on its usefulness in diagnosis, monitoring and different etiology of heart failure. Moreover, ANP is compared to the standard clinical biomarker BNP/NT-proBNP. Since for the latter much more data is available and detailed reviews already exist, it will only be comparatively referred to. Finally, analytical issues concerning general measurement of ANP as well as difficulties occurring in the context of pediatric blood sampling will be discussed.

## ANP in Pediatric Heart Failure

**Figure 1:**
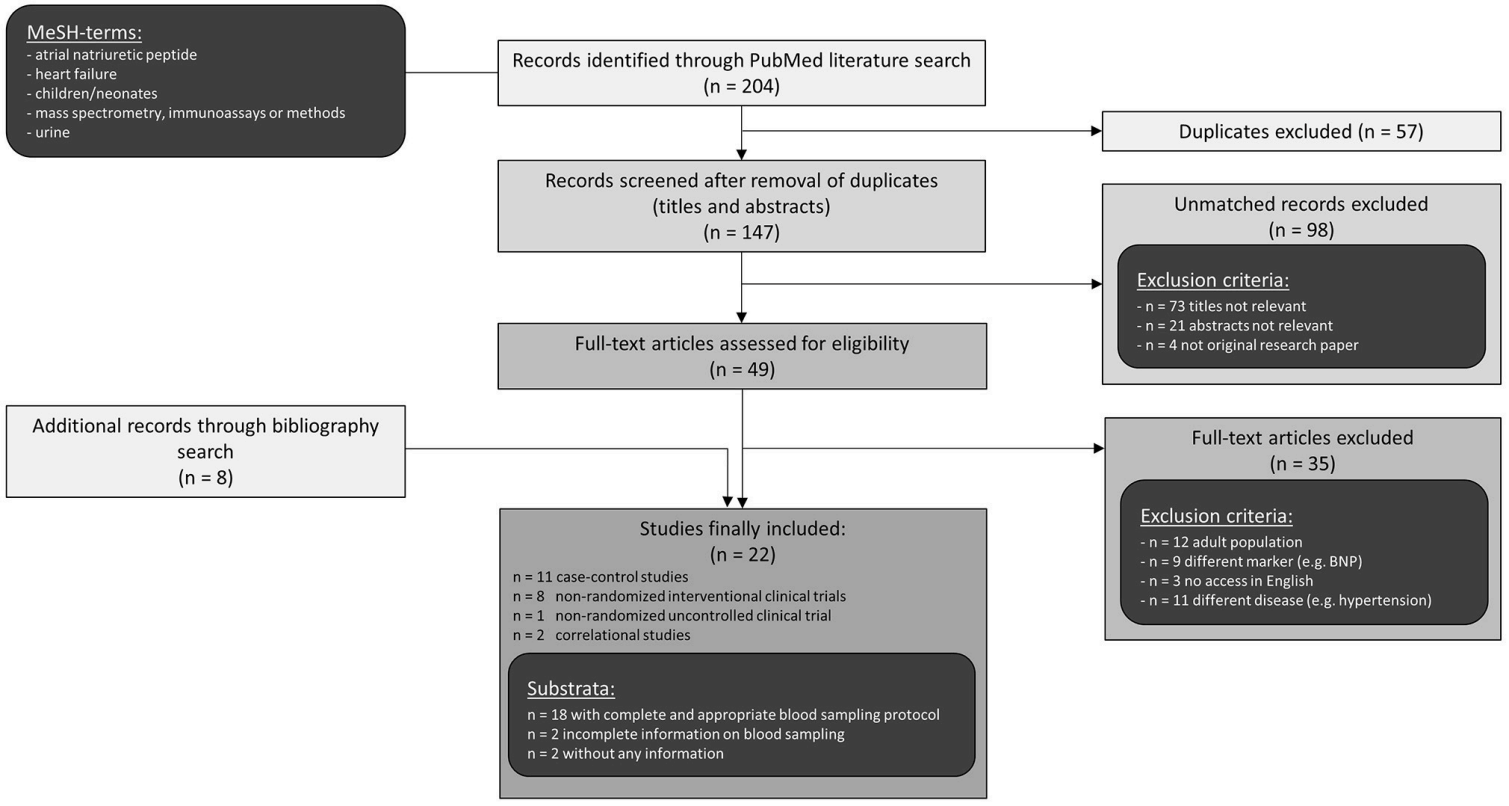
Literature search strategy.

A literature search using PubMed resulted in 147 studies of which 22 were classified as relevant and included in this review (Figure 1). Publications had to meet the following inclusion criteria: original research papers regarding pediatric population with congenital heart defect and/or heart failure and measurement of ANP, NT-proANP, or MR-proANP in blood or urine. Those criteria were fulfilled by 19 publications which reported pediatric ANP levels and are presented in Tables 1, 2. Another 3 studies determined MR-proANP or NT-proANP. All of them mainly evaluated the association between elevated ANP levels and diagnosis, severity, or etiology of heart failure as well as correlation with hemodynamic parameters, age or medical/surgical intervention.

**Table 1:** Overview of ANP levels in pediatric heart failure sorted in order of increasing age of diseased children in each section.

**Children**	**Age**	**n**	**ANP in heart failure (pg/mL)**	**ANP in controls (pg/mL)**	**Statistical operator**	**Blood sampling procedure**		**References**
**1. DIFFERENTIATION OF SYMPTOMATIC (S) AND ASYMPTOMATIC (A) CONGENITAL HEART DISEASE**								
Diseased	13.7 ± 14.1 mo	11	S: 232.5 ± 82.9^*^		mean ± SD	20 min supine Cubital or jugular vein Plasma	I	Agnoletti et al. (8)
	76.1 ± 49.4 mo	11	A: 48.4 ± 29.4^n/p^					
Control	Age-matched	30		24.1 ± 19.2				
Diseased	2.9 (0.3–16.7) y	23114	S: 285 (53.3–1990)^*^ A: 48.2 (8.6–449)^n/p^		median (range)	Awake Before procedures Peripheral veins Plasma	I	Westerlind et al. (9)
Control	1.1 (0.1–8.3) y	23		32.9 (11.7–212.2)				
Diseased	3.1 (0.3–16.2) y	61	S: 259.1 (86.4–246)^*^ A: 81.2 (31.8–164)^n/p^		median (range)	Awake Before procedures Peripheral veins Plasma	I	Holmgren et al. (10)
Control	1.1 (0.1–8.3) y	23		32.9 (11.7–212.2)				
Diseased	3.1 (0.3–13) y	59	S: Hepatomegaly: 116 ± 51 (*n* = 21)^*^ Respiratory retraction: 117 ± 37 (*n* = 17)^*^ Growth failure: 118 ± 58 (*n* = 17)^*^ A: Hepatomegaly: 72 ± 54 (*n* = 38)^n/p^ Respiratory retraction: 45 ± 43 (*n* = 28)^n/p^ Growth failure: 63 ± 47 (*n* = 42)^n/p^	n/a	mean ± SD	During cardiac catheterization Femoral vein Plasma	I	Suda et al. (11)
Diseased	4.3 ± 3.9 y	17	S: 748 ± 439^*^ A: 303 ± 71^n/p^	n/a	mean ± SD	Plasma Supine, quite for 20 min or 20 min after last dose of sedative during catheterization Femoral vein and right ventricle	IIb	Ross et al. (12)
Diseased	5.0 ± 1.1 y 6.6 ± 1.1 y	27 41	S: 35.5 ± 4.2^*^ A: 7.6 ± 0.6^n/p^		mean ± SD	>20 min resting Peripheral venous blood Plasma	I	Yeh et al. (13)
Control	3.5 ± 0.6 y	13		4.8 ± 1.1				
Diseased	5.2 (0.2–14.5) y	23 17	S: 284 (93–967)^n/p^ A: 57 (15–118)^n/p^		mean (range)	Supine >15 min Peripheral vein, after eating Plasma	IIa	Weil et al. (14)
Control	0.2–16 y	143		47 (2–109)				
Diseased	0.3–16 y	26	S: 303 (168–466)^*^ A: 42.9 (13.7–189)^n/p^	n/a	median (range)	Awake Before procedures Peripheral veins Plasma	I	Westerlind et al. (15)
**2. HEART FAILURE SEVERITY**								
Diseased	4.2 ± 1.1 y	14	NYHA I/II: 114.2 ± 47.3 (79% NYHA I)^*^		mean ± SD	Plasma Venous blood	I	Hayabuchi et al. (16)
Control	4.8 ± 0.9 y	n/a		18.6 ± 4.7				
Diseased	7.4 ± 3.0 y	22 23 3	NYHA I: 20 (5)^*^ NYHA II: 26 (6)^*^ NYHA III: 30 (8)^*^		median (IQR) resp. mean ± SD	Serum	III	Kotby et al. (17)
Control	7.8 ± 5 y	12		5.54 ± 1.41				
Diseased	15.2 ± 5.5 y	221 60 16	NYHA I: 45 ± 31^*^ NYHA II: 77 ± 49^*^ NYHA III: 125 ± 111^*^		mean ± SD	Plasma	III	Ohuchi et al. (18)
Control	n/a	51		19.1 ± 10				
**3. ETIOLOGIES**								
**(a) Acyanotic - left-to-right shunt**								
Diseased	22 (20–23) wk, gestational	8 7	VSD: 48.1 (30–315)^n.s.^ AVSD: 30 (12–76)^n.s.^		mean (IQR)	Fetal umbilical cord blood Plasma	IIa	Bartha et al. (19)
Control	21 (20.75–22) wk, gestational	14		29 (8.75–37.75)				
Diseased	4.8 (2.5–9.9) mo	5	VSD (*n* = 4), AVSD (*n* = 1): 175.1 (115–437)^n/p^	n/a	median (range)	Arterial catheter Plasma	I	Costello et al. (20)
Diseased	0.4 (9.3–4.5) y	11	VSD: 166 (31.8–346)^n/p^	n/a	median (range)	Awake Before procedures Peripheral veins Plasma	I	Westerlind et al. (15)
Diseased	2 (1–3) y 2 (0.1–15) y 6 (1–16) y	1/4 14/20 13	PDA: S: 416.0/A: 116.3 ± 26.5^*^ VSD: S: 350.6 ± 200.2^*^/A: 69.2 ± 36.5^*^ ASD: 99.4 ± 40.7^*^		mean ± SD resp. median (range)	Supine position at rest Peripheral vein	I	Kikuchi et al. (21)
Control	4 (0.1–15) y	30		44.6 ± 22.3				
Diseased	2.7 ± 3.3 y	12	VSD (*n* = 6), PDA (*n* = 4), AVSD (*n* = 2): 28.5 ± 6.6^*^		mean ± SD	Serum	III	Kotby et al. (17)
Control	7.8 ± 5 y	12		5.54 ± 1.41				
Diseased	5.0 (0.6–13.1) y	24	LRS with pulmonary hypertension: IVC: 198 (10–1417)^*^ PA: 456 (10–1697)^*^ AO: 336 (10–1190)^*^ RA: 360 (10–1655)^*^		mean (range)	Plasma	I	Oberhänsli et al. (22)
Control	6.2 (1.3–16.4) y	12		IVC: 56 (28–162) PA: 90 (46–147) AO: 78 (38–168) RA: 70 (35–122)				
Diseased	6.8 ± 6.4 y	6	PDA: 102.3 ± 30.3^*^		mean ± SD	Inferior vena cava during catheterization Plasma	I	Zeevi et al. (23)
Control	6.8 ± 2.5 y	9		24.6 ± 4.6				
Diseased	6.9 ± 1.1 y 5.0 ± 1.1 y	26 27	A: VSD (*n* = 15), ASD (*n* = 6), PDA (*n* = 4), PDA+ASD (*n* = 1): 8.3 ± 1.2^n.s.^ S: VSD (*n* = 16), ASD (*n* = 7), AVSD (*n* = 4): 35.5 ± 4.2^*^		mean ± SD	>20 min resting Peripheral venous blood Plasma	I	Yeh et al. (13)
Control	3.5 ± 0.6 y	13		4.8 ± 1.1				
Diseased	10.6 ± 3.6 y	14	ASD: 24 ± 9.8^*^		mean ± SD	Supine Peripheral vein Plasma	I	Muta et al. (24)
Control	6–18 y	10		17 ± 6.8				
Diseased	0.2–14 y	18 7 6	VSD: 221 ± 123^*^ ASD: 65 ± 42^n/p^ PDA: 124 ± 38^*^		mean ± SD	Peripheral vein Plasma	IIb	Matsuoka et al. (25)
Control	0.1–15 y	53		< 80				
**(b) Acyanotic - obstructive defects**								
Diseased	22 (20–23) wk, gestational	1	PS: 152^n.s.^		mean (IQR)	Fetal umbilical cord blood Plasma	IIa	Bartha et al. (19)
Control	21 (20.75–22) wk, gestational	14		29 (8.75–37.75)				
Diseased	8.5 ± 5.5 d	3 1	PS: 193.0 ± 61.6^n.s.^ AS: 192^n.s.^		mean ± SD	Inferior vena cava during catheterization Plasma	I	Zeevi et al. (23)
Control	14 ± 11 d	7		220.8 ± 16.2				
Diseased	4.5 (0.3–16.2) y	9	CoA: 42.2 (13.7–63.2) ^n/p^	n/a	median (range)	Awake Before procedures Peripheral veins Plasma	I	Westerlind et al. (15)
Diseased	6.2 ± 1.2 y	15	PS (*n* = 9), AS (*n* = 4), CoA (*n* = 2): 5.2 ± 0.7^n.s.^		mean ± SD	>20 min restingPeripheral venous blood Plasma	I	Yeh et al. (13)
Control	3.5 ± 0.6 y	13		4.8 ± 1.1				
Diseased	6.8 ± 6.4 y	168431	125.2 ± 15.8^*^ of which: PS: 126.4 ± 24.1^n/p^ AS: 141.7 ± 46.2^n/p^ CoA: 108.9 ± 42.5^n/p^ MS: 241^n/p^		mean ± SD	Inferior vena cava during catheterizationPlasma	I	Zeevi et al. (23)
Control	6.8 ± 2.5 y	9		24.6 ± 4.6				
Diseased	0.2–14 y	7	PS: 36 ± 21^n/p^		mean ± SD	Peripheral vein Plasma	IIb	Matsuoka et al. (25)
Control	0.1–15 y	53		< 80				
**(c) Cyanotic defects**								
Diseased	22 (20–23) wk, gestational	5321	Hypoplastic left heart: 15 (7.5–46)^n.s.^ Hypoplastic right heart: 44 (19–44)^n.s.^ TOF: 44 (19–44)^n.s.^ Univentricular heart: 53^n.s.^		mean (IQR)	Fetal umbilical cord blood Plasma	IIa	Bartha et al. (19)
Control	21 (20.75–22) wk, gestational	14		29 (8.75–37.75)				
Diseased	8.5 ± 5.5 d	2	ToGA: 344 ± 6.3^n.s.^		mean ± SD	Inferior vena cava during catheterizationplasma	I	Zeevi et al. (23)
Control	14 ± 11 d	7		220.8 ± 16.2				
Diseased	4 (0.1–15) y	10	TOF: 69.4 ± 38.8^n.s.^		mean ± SD resp. median (range)	Supine position at rest Peripheral vein	I	Kikuchi et al. (21)
Control	4 (0.1–15) y	30		44.6 ± 22.3				
Diseased	6.8 (1.8–14.7) y	21	TOF: IVC: 68 (10–169)^n.s.^ PA: 139 (10–262)^n.s.^ AO: 133 (10–365)^n.s.^ LA: 128 (24–210)^n.s.^ RA: 114 (10–308)^n.s.^		mean (range)	Plasma	I	Oberhänsli et al. (22)
Control	6.2 (1.3–16.4) y	12		IVC: 56 (28–162) PA: 90 (46–147) AO: 78 (38–168) RA: 70 (35–122)				
Diseased	0.2–14 y	7 5	TOF: 25 ± 11^n/p^ TA: 221 ± 90^n/p^		mean ± SD	Peripheral vein Plasma	IIb	Matsuoka et al. (25)
Control	0.1–15 y	53		< 80				
**(d) Dilated cardiomyopathy**								
Diseased	1.44 ± 1.4 y	12	29.25 ± 4.52^*^		mean ± SD	Serum	III	Kotby et al. (17)
Control	7.8 ± 5 y	12		5.54 ± 1.41				
Diseased	3.4 (0.3–14.8) y	6	412 (148–553)^n/p^	n/a	median (range)	Awake Before procedures Peripheral veins Plasma	I	Westerlind et al. (15)
**(e) Rheumatic heart disease**								
Diseased	13.2 ± 1.6 y 12.4 ± 2.9 y	12 12	S: 28.33 ± 5.78^*^ A: 26.5 ± 4.91^*^		mean ± SD	Serum	III	Kotby et al. (17)
Control	7.8 ± 5 y	12		5.54 ± 1.41				
**4. CARDIAC LOADING CONDITION**								
Diseased	4.5 (0.3–16.2) y 0.4 (9.3–4.5) y 3.4 (0.3–14.8) y	9 11 6	Pressure overload: 42.2 (13.7–63.2)^n/p^ Volume overload: 166 (31.8–346)^n/p^ LV dysfunction: 412 (148–553)^*^	n/a	median (range)	Awake Before procedures Peripheral veins Plasma	I	Westerlind et al. (15)
Diseased	3.7 (1.6–16.7) y 4.3 (0.3–16.2) y 1.0 (0.3–16.4) y 3.3 (0.3–15.0) y	35 29 60 13	No overload: 28.6 (8.6–105)^n.s.^ Pressure overload: 51.9 (8.7–210)^*^ Volume overload: 93.0 (15.9–346)^*^ Systolic ventricular dysfunction: 431 (43.8–1990)^*^		median (range)	Awake Before procedures Peripheral veins Plasma	I	Westerlind et al. (9)
Control	1.1 (0.1–8.3) y	23		32.9 (11.7–212.2)				
Diseased	6.8 (0.3–16.2) y 1.9 (0.3–14.7) y 0.5 (0.3–4.9) y 4.0 (0.5–13.3) y	15 11 16 18	LV pressure overload: 40.8 (12.6–219)^n.s.^ RV pressure overload: 69.3 (8.7–182)^*^ LV volume overload: 164 (31.8–346)^*^ RV volume overload: 57.2 (11.3–234.1)^*^		median (range)	Awake Before procedures Peripheral veins Plasma	I	Holmgren et al. (10)
Control	1.1 (0.1–8.3) y	23		32.9 (11.7–212.2)				

*A, asymptomatic; ANP, Atrial/A-type Natriuretic Peptide; AO, aorta; AS, aortic stenosis; ASD, atrial septal defect; AVSD, atrioventricular septal defect; CoA, Coarctation of the aorta; d, days; IQR, interquartile range; IVC, inferior vena cava; LA, left atrium; LRS, left-to-right shunt; LV, left ventricle; min, minutes; mo, months; MS, mitral stenosis; n, number; n/a, not available; NYHA, New York Heart Association; PA, pulmonary artery; PDA, patent ductus arteriosus; PS, pulmonary stenosis; RA, right atrium; RV, right ventricle; S, symptomatic; SD, standard deviation; TA, Tricuspid atresia; TOF, tetralogy of Fallot; ToGA, transposition of great arteries; VSD, ventricular septal defect; wk, weeks; y, years*.

* *Statistically significant to reference group/controls*,

*^n/p^ significance not provided, ^n.s.^ non-significant*.

*Sample collection protocol*:

*I: detailed information available regarding: blood sampling procedure, used inhibitors, storage conditions*.

*IIa: detailed information available regarding: blood sampling procedure, storage conditions; incomplete information regarding used inhibitors*.

*IIb: detailed information available regarding: blood sampling procedure, used inhibitors; incomplete information regarding storage conditions*.

*III: without any information regarding: blood sampling procedure, used inhibitors, storage conditions*.

**Table 2:** ANP levels influenced by medical or surgical intervention sorted in order of increasing age.

**Intervention**	**Age**	**n**	**ANP before intervention (pg/mL)**	**ANP after intervention (pg/mL)**	**ANP in controls (pg/mL)**	**Statistical operator**	**Blood sampling procedure**		**References**
Catheterization	8.5 ± 5.5 d	6	243.0 ± 42.1	30 min: 243.6 ± 48.4^*^ FU: 62.1 ± 12.7^*^		mean ± SD	IVC during catheterization Plasma	I	Zeevi et al. (23)
None	14 ± 11 d	7			220.8 ± 16.2				
Catheterization	6.8 ± 6.4 y	22	125.2 ± 15.8	30 min: 75.6 ± 11.4^*^ FU: 42.9 ± 5.0^*^					
None	6.8 ± 2.5 y	9			24.6 ± 4.6				
Cardiopulmonary bypass	4.8 (2.5–9.9) mo	5	175.1 (115–437)	1 day: 44 (28–81)^*^	n/a	median (range)	Arterial catheter Plasma	I	Costello et al. (20)
Cardiac surgery	41.11 ± 25.39 mo	27	Normal PBF: 175.9 ± 253.2	24 h: 149.3^n.s.^ 48 h: 155.8^n.s.^		mean ± SD	15 min supine 9:00 am Before surgery: peripheral vein After surgery: central venous catheter Plasma	I	Alvarez Kindelan et al. (26)
	30.61 ± 40.74 mo	38	High PBF: 229.9 ± 311.2	24 h: 183.8^n.s.^ 48 h: 187.2^n.s.^					
None	0.2–14 y	48			46.5 ± 25.5				
Catheterization	3.1 (0.3–13) y	59	99.8 ± 60.2	8 mo: 23.8 ± 10.1^*^	n/a	mean ± SD	During cardiac catheterization Femoral vein Plasma	I	Suda et al. (11)
Right venricular angiography	6.8 (1.8–14.7) y	21	TOF before: IVC: 68 (10–169) PA: 139 (10–262) AO: 133 (10–365) LA: 128 (24–210) RA: 114 (10–308)	After: IVC: 217 (42–612)^*^ PA: 33 (49–805)^*^ AO: 360 (95–910)^*^ LA: 228 (77–525)^*^ RA: 298 (21–875)^*^		mean (range)	Plasma	I	Oberhänsli et al. (22)
	5.0 (0.6–13.1) y	24	LRS with pulmonary hypertension before: IVC: 198 (10–1417) PA: 456 (10–1697) AO: 336 (10 −1190) RA: 360 (10–1655)	After: IVC: 412 (63–1568)^*^ PA: 883 (74–3220)^*^ AO: 760 (116–1960)^*^ RA: 594 (45–2275)^*^					
Right venricular angiography	6.2 (1.3–16.4) y	12		Controls after: IVC: 181 (70–346)^*^ PA: 207 (105–266)^*^ AO: 221 (66–567)^*^ RA: 260 (122–378)^*^	Before: IVC: 56 (28–162) PA: 90 (46–147) AO: 78 (38–168) RA: 70 (35–122)				
Transcatheter closure of ASD	10.6 ± 3.6 y	14	24 ± 9.8	5 min: 34 ± 18^*^ 24 h: 19 ± 11^n.s.^		mean ± SD	Supine Peripheral vein Plasma	I	Muta et al. (24)
None	6–18 y	10			17 ± 6.8				
Captopril 0.5 mg/kg	13.2 ± 1.6 y 12.4 ± 2.9 y 2.7 ± 3.3 y 1.44 ± 1.4 y	12 12 12 12	RHDF: 28.33 ± 5.78 RHD: 26.5 ± 4.91 CHD: 28.5 ± 6.6 DCM: 29.25 ± 4.52	RHDF: 15.3 ± 5.3^*^ RHD: 10.7 ± 2.5^*^ CHD: 11.5 ± 3.8^*^ DCM: 15.7 ± 10.7^*^		mean ± SD	Serum	III	Kotby et al. (17)
None	7.8 ± 5	12			5.54 ± 1.41				

*ANP, Atrial/A-type Natriuretic Peptide; AO, aorta; ASD, atrial septal defect; CHD, congenital heart disease; d, days; DCM, dilated cardiomyopathy; FU, follow-up after 7-30 days; h, hours; IVC, inferior vena cava; LA, left atrium; LRS, left-to-tight shunt; min, minutes; mo, months; n, number; n/a, not available; PA, pulmonary artery; PBF, pulmonary blood flow; RA, right atrium; RHD, rheumatic heart disease; RHDF, rheumatic heart disease in failure; SD, standard deviation; TOF, tetralogy of Fallot; y, years*.

* *statistically significant decrease through intervention*,

*^n.s.^ non-significant*.

*Sample collection protocol*:

*I = detailed information available regarding: blood sampling procedure, used inhibitors, storage conditions*.

*III = without any information regarding: blood sampling procedure, used inhibitors, storage conditions*.

Heart failure in children has a different etiology compared to adults. While in adulthood coronary artery diseases and hypertension are usually causative, it is cardiomyopathies (CMP) and congenital heart defects (CHD) in infants. While CMP leads to low output cardiac failure, CHD results in high output cardiac failure. Less common and more present in developing countries is heart failure originating from rheumatic heart disease (RHD) (4). It is possible that depending on the location of the cardiac malformation, differences in the diagnostic performance of natriuretic peptides arise (15).

### ANP as a Diagnostic Marker in Pediatric Heart Failure

In heart failure, ANP secretion is extended from atria to ventricles. ANP can reflect changes faster since it is stored as a prohormone, whereas BNP has to be genetically translated before release (22, 27–29). ANP can indicate acute volume overload and hemodynamic changes, while BNP better reflects prolonged overload and shows increased stability (30). Nevertheless, ANP and BNP significantly correlate with each other in adults as well as in children, with ANP presenting higher levels than BNP (11, 15, 18).

#### Identifying Children With Heart Failure

Concerning the diagnosis of heart failure, ANP levels are significantly increased in diseased children compared to healthy controls (15, 26, 31). In children with dilated cardiomyopathy (DCM), CHD or RHD, ANP can differentiate symptomatic (heart failure) and asymptomatic children (e.g., mean 232.5 pg/mL vs. 48.4 pg/mL) (8–15).

In asymptomatic infants with CHD, levels of ANP compared to controls are elevated, but not necessarily significantly (e.g., 48.4 pg/mL vs. 24.1 pg/mL) (8, 13, 18). In children with ventricular septal defect (VSD), ANP, and BNP appeared to be comparable markers to identify children suffering from VSD. ANP displayed higher sensitivity, but lower specificity in identifying those with significant shunt or pulmonary hypertension (11). For further details, please see Table 1.1.

In the case of NT-proANP, Holmstrom et al. reported that CHD patients had significantly elevated serum levels (median 904 pmol/L vs. median 384 pmol/L) (32). In 2000, the same authors published higher level data, speaking of a 10-fold increase in heart failure patients (mean 4515 pmol/L) in comparison to controls or other diseases (urologic or malignant) (33). Samples were also measured with an in-house immunoassay but plasma instead of serum was used.

Similarly to ANP and NT-proANP, the marker MR-proANP can be utilized to diagnose heart failure in children with CHD and CMP (34). Median serum MR-proANP in healthy children was 40 pmol/L. In CHD patients levels increased up to 72.8 pmol/L and in CMP up to 76.2 pmol/L.

In comparison to NT-proBNP, and in contrast to adults, MR-proANP was slightly inferior regarding diagnostic accuracy (34, 35).

Altogether, ANP, NT-proANP, and MR-proANP can like BNP be used to separate children with heart failure from those without. Regarding asymptomatic pediatric CHD, ANP is equally to BNP not necessarily significantly elevated compared to healthy controls (36). Only 3 studies concerning NT-proANP/MR-proANP measurement in pediatric heart failure have been conducted, consequently further research needs to clarify their relevance and possible benefits in children.

#### Classification of Pediatric Heart Failure Severity

Levels of ANP cannot only serve for the diagnosis of heart failure but further correlate with the severity of heart failure. They increase stepwise with the New York Heart Association (NYHA) stage, irrespective of the underlying cause (Table 1.2, e.g., mean: NYHA I: 45 pg/mL, II: 77 pg/mL, III: 125 pg/mL) (17, 18). The same applies to NT-proANP which correlates positively with the severity of heart failure (32). There is no data for MR-proANP.

Thus, ANP and NT-proANP discriminate equally to BNP/NT-proBNP between different stages of heart failure severity (37–40).

#### Discrimination of Cardiac Load in Pediatrics

ANP is released in response to cardiac wall stretch from the atrial tissue. Consequently, in CHD, ANP correlates with right and left atrial pressure, mean pulmonary arterial pressure and pulmonary resistance (8, 22, 26, 41). Hayabuchi et al. observed a correlation between ANP and right ventricular pressure, but only BNP correlated significantly with right ventricular volume (16).

When comparing elevated ANP levels resulting from different etiologies of heart failure, the highest were observed in systolic ventricular dysfunction (median: 431 pg/mL) followed by volume overload with preserved systolic dysfunction (median: 93.0 pg/mL). Pressure overload showed the lowest increment (median: 51.9 pg/mL), whereas the group without overload (median: 28.6 pg/mL) did not differ from controls (median: 32.9 pg/mL) (9, 15). Also, significantly higher ANP and BNP levels could be observed in left ventricular volume overload (median: 164 pg/mL) compared to right ventricular volume overload (median: 57.2 pg/mL) or pressure overload (median left: 40.8 pg/mL, right: 69.3 pg/mL) (10). Kotby et al. confirmed that in left ventricular volume overload resulting from different etiologies, levels of ANP are significantly elevated compared to controls (17). Detailed levels are displayed in Table 1.4.

ANP can discriminate cardiac loading conditions equally to BNP showing the same tendencies in the different conditions (42). However, BNP might be superior to ANP in the right ventricular system since only BNP has been shown to significantly correlate with right ventricular volume so far.

#### Differentiation of Underlying Etiology

As previously mentioned, etiological factors attributable to pediatric heart failure have been identified as DCM, RHD and CHD.

Pediatric patients with DCM present with the highest levels of ANP and MR-proANP—fully in line with the BNP data (Table 1.3d) (9, 15, 17, 34, 43). The hypothesis of a resistance to natriuretic peptide effects in patients with DCM has been proposed, additionally to an increased ANP synthesis and secretion, which accounts for the highest observed levels.

In RHD, ANP levels are significantly higher than in controls. The values are in the range of other etiologies like DCM and CHD (Table 1.3e) (17).

Most studies have focused on CHDs. Those can be categorized into cyanotic [e.g., tetralogy of Fallot (TOF), tricuspid atresia (TA)] and acyanotic defects. In the latter, obstructive defects [e.g., pulmonary stenosis (PS), aortic stenosis (AS), coarctation of the aorta (CoA), mitral stenosis (MS)] and left-to-right shunts [e.g., VSD, atrioventricular septal defect (AVSD), atrial septal defect (ASD), patent ductus arteriosus (PDA)] are further differentiated. ANP levels in each group can be seen in Tables 1.3a,b,c.

Levels in VSD, PDA, or TA are significantly elevated compared to other malformations like CoA, ASD or TOF and also to controls (15, 21, 23, 25). It further has been shown that in VSD, ANP as well as BNP correlate with the shunt size of the defect [ANP: small: 59.1 ± 28.9 pg/mL (*n* = 17), moderate: 130.6 ± 12.2 pg/mL (*n* = 5), large: 386 ± 194.1 pg/mL (*n* = 12)] (11, 21).

Significantly elevated levels of ANP compared to controls in ASD were measured by Muta et al. whereas Yeh et al. only reported increased levels in symptomatic children but pooled children with VSD, ASD, AVSD, and PDA (13, 24). In TOF patients, Oberhänsli et al. and Kikuchi et al. observed insignificantly elevated levels of ANP in comparison to controls, whereas Matsuoka et al. measured levels in the range of controls (21, 22). While Zeevi et al. reported a significant increase of ANP in PS, AS, CoA and MS compared to controls, Matsuoka et al. and Yeh et al. could not confirm this (13, 23, 25). By examining whether fetuses with cardiac malformations already show increased ANP levels, different types of CHD did not significantly differ (19). Furthermore, no significant difference to fetal controls could be detected. NT-proBNP can identify fetal cardiac defects but the results may be influenced by a higher mean age of 6 weeks (44).

Only one study assessed pediatric NT-proANP levels regarding the etiology and stated highest levels in AVSD followed by PDA > AS > ASD > VSD (32). These results contrast to measurements of NT-proBNP or ANP, where higher concentrations are reported in VSD and PDA than in ASD (32, 45).

In conclusion, DCM, RHD and CHD show elevated levels of ANP as it is seen for BNP. Nevertheless, ANP levels are inhomogeneous if different types of CHDs are compared. This might be attributed to the small and heterogeneous pediatric study populations. Generally, more complex CHDs present higher levels of ANP, in line with measurements of BNP (42).

### Monitoring the Cardiac Status With ANP in Pediatric Heart Failure

ANP levels decreased after treatment with the Angiotensin-Converting-Enzyme inhibitor captopril in patients with left volume overload, reflecting the improvement of NYHA stage and left ventricular remodeling (Table 2) (17). Infusion of the short-acting sympathomimetic dobutamine given to children with surgically repaired TOF decreased ANP and BNP significantly from elevated levels. Larger changes in ANP reflect atrial and ventricular pressure changes and the larger decline of ANP might be attributed to its faster release and clearance compared to BNP. After stopping the infusion, levels rose back to the baseline (16).

Regarding surgical interventions in children with CHD, interventional catheterization significantly lowered preoperative values already 30 min subsequent to the operation and further after 7–30 days (Table 2). The ANP levels stayed elevated compared to age-matched controls (23). Setting the follow-up after a longer period, in infants with VSD, preoperative ANP and BNP levels also decreased significantly compared to measurement at 8 months postoperative (11). Cardiopulmonary bypass in children with a left-to-right shunt in failure resulted in a decrease of baseline ANP levels one day after. Surprisingly, in the same population BNP levels were normal at baseline and rose during operation (20). In 14 children undergoing transcatheter closure of ASD, significantly elevated levels of ANP compared to controls were reported before closure (24). Those increased significantly 5 min after closure, but declined within 24 h to levels insignificantly different from controls, maintaining these levels during follow-up at 1 and 3 months. Contrastingly, BNP showed a prolonged elevation. At the time-point of 24 h ANP levels were higher than preoperative or 5 min after operation. The authors suggested that this might be reflective of the mechanistic differences of the atria and the right ventricle as compared to the left ventricle during the remodeling process after correction.

Cardiac surgery of various CHDs in 65 children with significantly increased baseline levels of ANP compared to controls, resulted in a non-significant decrease of ANP at 24 and 48 h post-operative (26). Details of each intervention are presented in Table 2.

In conclusion, anatomical correction of malformations, as well as medical treatment of heart failure, reduce ANP levels in children. Differences between ANP and BNP can be seen due to their diverging secretion and clearance reflecting the faster acting of ANP.

### Effect of Age and Body Weight on ANP Levels in Healthy Infants

There is conflicting data on the correlation of ANP and age. Klar et al. obtained significantly different levels between the age groups of 3 months to 3 years on the one hand and 3 to 14 years on the other hand. Ohuchi et al. and Matsuoka et al. also observed an inverse correlation in control subjects from 1 to 40 years, respectively 1 month to 15 years. (18, 25, 46).

Few studies included neonates, in which levels of ANP were significantly elevated. But beyond neonatal period up to adulthood, these levels did not vary significantly (14, 23). Hemodynamic changes after birth are being held responsible for increased natriuretic peptide levels (14, 23, 47).

By contrast, there are also some studies that measured ANP in healthy children older than 1 month without observing any correlation (8, 9, 41, 48). In comparison, BNP is high during the first days of life, decreases rapidly soon afterward and slowly during the remaining period of childhood (49).

In case of NT-proANP and MR-proANP, significantly negative connections to age were seen (32–34). NT-proANP levels are significantly elevated during the first days of life with a peak on the first day reaching a more pronounced increase than NT-proBNP but displaying constant levels within few days (47). Other studies observed the highest levels of NT-proANP in newborns until 1 month of age, passing into slightly elevated levels during the first year of life and subsequently resulting in consisting ones (32, 33). In case of pro-ANPs, not only adapting mechanisms of the heart are causative, but also—due to their renal clearance mechanism—the changing of the glomerular filtration rate with age (33). However, only in children with severe renal dysfunction (glomerular filtration rate < 30–40 mL/min/1.73 m^2^) did increased levels of NT-proANP occur and thus the efficiency in terms of diagnosing heart failure is usually not influenced (32, 33).

In conclusion, levels of ANPs in children are relatively constant after passing the peak caused by adaptional processes after birth, equivalently to BNPs. Following from these peak levels, ANP concentrations in newborns with pulmonary stenosis and aortic stenosis did not significantly differ from levels in controls as already physiologically high levels of ANP can mask the elevation induced by heart failure (Table 1.3b) (23). In neonates with transposition of great arteries, ANP levels were elevated (Table 1.3c). Therefore, the use of ANP and generally natriuretic peptides in neonates in a diagnostic manner has to be carefully applied.

Furthermore, ANP and BNP significantly negatively correlated with bodyweight in connection with symptoms of heart failure (11). Therefore, infants with growth failure, a clinical sign of heart failure, present also higher levels of ANP (11, 50).

### Meaningful Alternatives to Blood Sampling

The European Medicines Agency demands considering non-invasive alternatives to blood sampling whenever possible for pediatric studies (51).

#### Urine

ANP is found in urine as well and was determined in children to assess the possibility of circumventing stressful blood sampling in children (8). But ANP was not detectable in healthy controls (*n* = 30). Urine levels did not correlate with plasma levels in CHD patients (*n* = 22). Further, it was not possible to distinguish patients with (*n* = 11, mean 13.7 months) and without clinical signs (*n* = 11, mean 76.1 months) by the means of urinary ANP. As the main metabolism of ANP is not organ-specific (e.g., kidney), measurement of ANP in urine cannot make up as an alternative to plasma measurement. Hence, NT-pro-peptides which are renally excreted could rather be an alternative, but currently there is no data for NT-proANP, NT-proBNP, or also BNP in urine in pediatric heart failure.

#### Saliva

In healthy men, ANP can be measured in saliva which correlates with plasma levels (52). So far, no studies evaluated the significance of salivary ANP in heart failure, but for NT-proBNP and BNP first studies were conducted. Those indicate that adult heart failure patients present higher levels than controls (53, 54). Saliva offers the advantages of being non-invasive, less demanding and easier to handle than blood. Thus, further research should be conducted since it seems to offer a promising alternative approach to the determination of biomarkers in heart failure, an approach which would be especially advantageous in pediatrics.

## Preanalytical and Analytical Issues During Measurement of ANP

### Collection and Processing of Blood Samples

ANP has a short half-life (2–5 min) and has to be treated carefully after blood sampling (55). Despite its short half-life, it is proven that ANP with EDTA and aprotinin is stable for 2 months at −20°C and for 2 h at room temperature which allows for analysis (56, 57). Plasma should be obtained as fast as possible as ANP clearance receptors are located on platelets and therefore concentrations in whole-blood diminish (58). Hemolyzed samples should be excluded as falsely low concentrations of ANP will be measured (59). However, hemolytic samples can be used for the determination of NT-proANP (60, 61).

### Difficulties Concerning Pediatric Sampling

The position of the patient during blood sampling affects ANP levels substantially. Moving into supine position causes levels of ANP to rise, probably as a consequence of venous return and the following increment in atrial pressure (62–64). In severe heart failure, a change from supine to upright position is associated with further increase of supine ANP levels, likely caused by tachycardia or increased sympathetic nervous activity (62). Therefore also crying, which is common in infants during venipuncture, is most likely influencing levels of ANP and also excitement may play a role. Further the site of blood collection matters because central and peripheral plasma concentrations differ (65). Thus, a well-standardized protocol is needed and deviations have to be recorded in order to be able to interpret findings adequately.

## Conclusion

ANP serves as a helpful marker for the diagnosis of pediatric heart failure and follow-up of treatment and after operation in children. Due to its fast release, ANP offers the advantage of displaying rapid changes during therapy or operation. Nevertheless, it altogether offers no major advantage over BNP and NT-proBNP. The perspective of establishing ANP as a standard clinical biomarker for diagnosis and prognosis seems to be low, but it has high potential in research for a better understanding of the natriuretic peptide system and the impact of drugs (e.g., sacubitril/valsartan). So far, no measurement of ANP/NT-proANP has been reported in humans taking sacubitril/valsartan. Thus, it remains to be seen whether it turns out to be an alternative. ANP is susceptible to a lot of preanalytical and analytical issues, especially in pediatric sampling. Finally, saliva is suggested to be a promising alternative to blood sampling enabling non-invasive measurement of ANP.

## Author Contributions

Conception and design of the work was developed in close collaboration by TG and BB. Data collection and analysis was performed by TG. The interpretation and drafting of the article was done by TG and BB. Critical revision and final approval of the version to be published was given by BB.

### Conflict of Interest Statement

The authors declare that the research was conducted in the absence of any commercial or financial relationships that could be construed as a potential conflict of interest.

## Abbreviations

ANP: Atrial/A-type Natriuretic Peptide
AS: aortic stenosis
ASD: atrial septal defect
AVSD: atrioventricular septal defect
BNP: Brain/B-type Natriuretic Peptide
CHD: congenital heart defect
CMP: cardiomyopathy
CoA: Coarctation of the aorta
DCM: dilated cardiomyopathy
EDTA: ethylenediaminetetraacetic acid
MR-proANP: mid-region Atrial/A-type Natriuretic Peptide
MS: mitral stenosis
NT-proANP: N-terminal Atrial/A-Type Natriuretic Peptide
NT-proBNP: N-terminal Brain/B-type Natriuretic Peptide
NYHA: New York Heart Association
PDA: patent ductus arteriosus
PS: pulmonary stenosis
RHD: rheumatic heart disease
TA: tricuspid atresia
TOF: tetralogy of Fallot
VSD: ventricular septal defect.
